# The switching of strong spin wave beams in patterned garnet films

**DOI:** 10.1038/s41598-017-06531-2

**Published:** 2017-08-18

**Authors:** R. Gieniusz, P. Gruszecki, M. Krawczyk, U. Guzowska, A. Stognij, A. Maziewski

**Affiliations:** 1 Faculty of Physics, University of Białystok, Ciołkowskiego 1L, 15-245 Białystok, Poland; 2 Faculty of Physics, Adam Mickiewicz University in Poznan, Umultowska 85, 61-614 Poznań, Poland; 3 Scientific-Practical Materials Research Center at National Academy of Sciences of Belarus, P. Brovki 19, Minsk, 220072 Belarus

## Abstract

The application of spin waves in communication with information encoded in amplitude and phase could replace or enhance existing microelectronic and microwave devices with significantly decreased energy consumption. Spin waves (SW) are usually transported in a magnetic material shaped to act as a waveguide. However, the implementation of SW transport and switching in plane homogeneous magnetic films and running as a narrow beam with a small divergence angle still present a challenge. We propose a realization of a strong SW switchers based on a patterned yttrium iron garnet (YIG) film that could serve as a magnonic fundamental building block. Our concept relies on the creation of a narrow beam of relatively short-wavelength SW by effect of a total non-reflection, found to be tied to refraction on the decreasing internal magnetic field, near a line of antidots at YIG. Nonreciprocal SW excitation by a microstrip antenna is used for controlling the direction of the signal flow. We demonstrate unique features of the propagation of microwave-excited SW beams, provide insight into their physics and discuss their potential applications in high-frequency devices.

## Introduction

The conventional CMOS technology has limitations in terms of operational speed, miniaturization and power consumption; these limitations are related to the motion of electrons^1–4^. Therefore, a major stream in current research is aimed at the development of concepts alternative to CMOS, in which the transport of charges would be replaced by that of quasiparticles^5–8^, including spin waves (SWs)^9–12^. SWs have been studied for years^13^ because of their interesting fundamental physics and potential applications in high-frequency devices^14^. Micrometer-thick films of yttrium iron garnet (YIG) have been used in tunable bandpass filters, circulators, SW isolators, delay lines and diodes in the GHz frequency range^15–19^. Yu *et al*.^20^ have recently succeeded in propagating SWs in an only 20 nm thick YIG film. However, propagating SWs as a narrow beam with a well-defined direction, remain unexploited from the point of view of applications. That is despite the increasing progress in last years showing a possibility for generating^21–23^ and steering SWs with inhomogeneity of the magnetic field^24^, film thickness^25, 26^ and magnetization orientation^27^.

Magnetostatic surface SWs (MSSWs)^28^ have a number of interesting properties: a nonlinear dispersion relation and isofrequency dispersion relation lines (IFDRLs) with linear parts^29^; nonreciprocity^30–32^, and sensitivity to magnetic field direction in terms of propagation direction and its localization; and a dispersion that can be controlled by a magnetic field or by adjusting the geometry and pattern of the medium^33, 34^. In a homogeneous film, zero curvature points and segments in IFDRLs lead to the occurrence of caustic SWs^35^ which can be generated by both active^36, 37^ and passive^38^ point excitation sources. The properties of IFDRLs of MSSWs allow also for existence of a phenomenon known in literature as *total non*-*reflection* ^29, 34^ which arises for SWs incident at specific angles on the reflective boundary, oriented under critical angle with respect to the external magnetic field. Total non-reflection can result in the generation of a SW beam^39, 40^, the effect further explored and exploited in our study towards applications^3^.

In the paper we propose and experimentally demonstrate, by Brillouin light scattering (BLS) technique, a simple realization of a strong SW beam switchers on patterned YIG film. This could serve as a basic element of microwave devices used to control signals by change of the propagation direction of signal flowing. We applied the total non-reflection effect on only a few of antidots in a plane YIG film to create a strong SW beam. To switch the direction of the beam we used the effect of non-reciprocal excitation of the surface spin waves by the microstrip antenna^18, 41^. Moreover, we demonstrate by the micromagnetic simulations (MS) and iso-frequency curve method that the formation of a strong SW beam results from the refraction of SWs near the antidots in an area in which the internal magnetic field is reduced by the demagnetizing field.

## Results

### Sample characteristics and geometry

**Figure 1:**
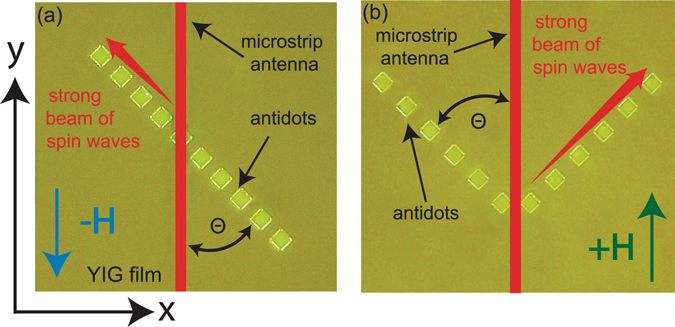
Photos showing the structures under investigation and demonstration principles of the two spin wave switchers. (**a**) 180° switcher, and (**b**) 90° V-type geometry switcher. Microstrip antenna (red vertical bar) placed on the surface of a YIG film under magnetic field **H** parallel to the antenna, and at the angle θ = 45° with respect to the line of square antidots. Microwaves in the antenna induce SWs propagating in the sample to the left (**a**) or right (**b**) with the field **H** in the negative or positive direction of the *y*-axis, respectively.

The studied samples were 4.5 and 2.8 µm thick monocrystalline YIG films epitaxially grown along the (111) crystal axis on transparent gadolinium gallium garnet substrates. Estimated by ferromagnetic resonance measurements, the Gilbert damping factor was α = 0.0001. The samples were patterned by chemical etching to produce a single line of 50 µm × 50 µm antidots with a 50 µm spacing (Fig. 1a) or a V-shaped pattern of two lines of antidots (Fig. 1b) in thicker and thinner YIG films, respectively. SWs were excited by a 30 µm wide microstrip antenna lying on the top of the sample and supplied by a microwave generator operating in continuous mode and the power limited to 5 dBm, low enough to avoid nonlinear effects.

### Experimental demonstration

A static magnetic field **H** was parallel to the microwave antenna oriented along the *y*-axis creating angle θ (=45°) with the lines of antidots. This angle corresponds to the critical angle θ_cr_ for total non-reflection^34^ for magnetic field amplitude 980 Oe. The antenna excited SW with wavevector **k** oriented along the *x*-axis. SW interaction with the lines of antidots was studied using a BLS spectrometer in the reflection configuration^42^. A probe solid-state laser beam with a wavelength of 532 nm was scanned across the sample with 0.015 mm step, the BLS intensity *I* is proportional to the square of the dynamic magnetization amplitude^38, 40^.

**Figure 2:**
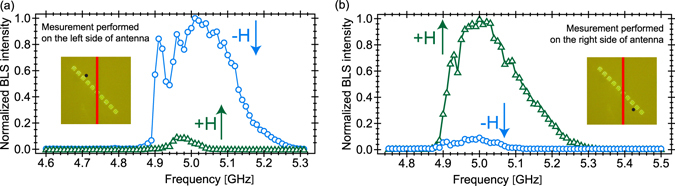
Experimental demonstration of the switching of spin waves with change of the magnetic field orientation. Normalized BLS intensity spectrum as a function of frequency, measured on the left (**a**) and right (**b**) of the microwave antenna for a laser light spot (black dot in the insets) near the right and left side of the antidots, with two magnetic field orientations, +**H** and −**H**, respectively, at *H* = 980 Oe.

**Figure 3:**
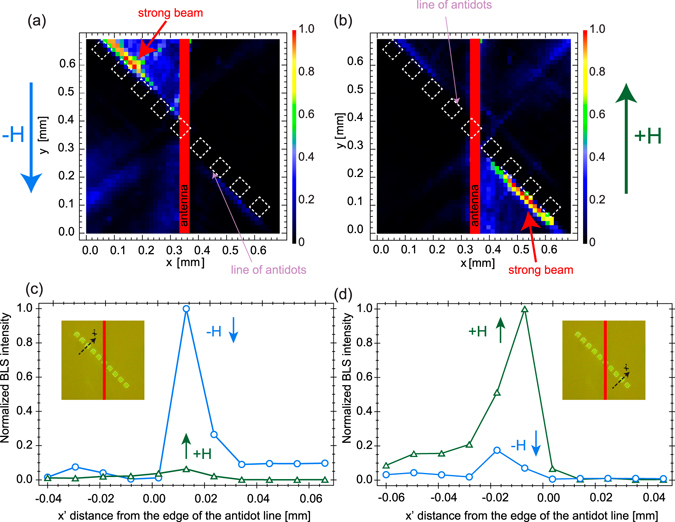
Experimental demonstration of the spin wave beam formation combined with a spin wave switching. Two-dimensional color map of the SW amplitude based on the acquired BLS data. A SW beam is formed along the antidot line, and switched by 180° on reversal of the magnetic field direction from −**H** (**a**) to +**H** (**b**). The SWs are generated by a microwave antenna (red vertical bar) at a frequency of 4.98 GHz at *H* = 980 Oe. (**c**,**d**) Spatial distribution of normalized BLS intensity along a line perpendicular to the row of antidots (black-dashed line in the insets) on the left (**c**) and right (**d**) of the antenna for −**H** (blue circles) and +**H** (green triangles). The zero distance corresponds to the edge of the antidot.

Let us consider possibility of strong SWs beam 180° switcher. Combined nonreciprocal SW excitation by a microwave antenna^15, 18, 43, 44^ and total non-reflection effects^38, 40^ were studied locally for different excitation frequencies in two small areas, selected by focused light spot, near the antidots, see the black dot in the insets in Fig. 2. The BLS amplitude-frequency characteristics were measured for two orientations of the magnetic field, +**H** and −**H**. For the light spot located at the left side of the antenna (Fig. 2(a)), the BLS intensity is significantly (about 10 times) larger for the magnetic field direction −**H** than +**H**. Measurements performed on the region at the right of the antenna (Fig. 2(b)) show a reverse situation^44^. SW of frequency 4.98 GHz interaction with the antidots lines is presented at wider sample area in Fig. 3(a),(b) which illustrate two-dimensional BLS maps of SW intensity *I*(*x*, *y*) distribution for the field directions −**H** and +**H**, respectively. Figure 3(a) shows the strong SW beam of propagating left-up along the line of antidots for −**H**. The SW beam travels right-down along the antidots line while reversing magnetic field to +**H**. The SW beams profiles are presented in Fig. 3(c),(d), they show BLS intensity *I*(*x*′) cross-scans along two lines perpendicular to the antidots edge (see insets at Fig. 3c,d) for both +**H** and **−H**. Around 15 ± 5 μm the strong beams width can be deduced from these scans. These beams maxima are shifted from the antidots edge by about 10 ± 5 μm. Thus, we have experimentally demonstrated 180° switcher showing the possibility of creation, along the antidots line, strong SW beams which propagation direction could be 180° changed while reversing the magnetic field direction.

**Figure 4:**
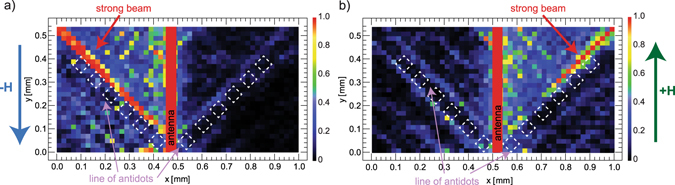
Experimental demonstration of the SWs beam switching property with the change of the magnetic field direction in the V-type geometry. (**a**) and (**b**) Two-dimensional BLS p﻿lots mapping the intensity of the SWs in switching between the beams propagating at +45° and −45° angle by change of the magnetic field direction from −**H** to +**H**.

Another solution of strong SW beam 90° switcher is illustrated in Fig. 4 where the microwave antenna (parallel to the external magnetic field **H**) crosses the V-type antidots lines. The SW beam propagation direction was changed by 90° while reversing the magnetic field direction. Due to thinner YIG film the optimal frequency here is decreased to 4.7 GHz as compared to the previous sample. Interestingly, we observed in both samples that the accumulated intensity of the refracted SWs beam increases further from the microstrip line it is measured, pointing that accumulation of the SWs amplitude in this process exceeds decrease of the amplitude resulting from an SWs’ damping.

## Discussion

To explain effect of creation of strong SW beam we consider local demagnetization field, driving changes of SW refraction while approaching an edge of the antidots line. The demagnetizing field gradually decreases the internal magnetic field, and thus, shifts the SWs dispersion relation in the frequency scale. For propagating SWs the changes of the dispersion can be described by a change of the refractive index. For SWs we will use refractive index defined (similar as in optics) as a ratio of the phase velocities $v$ of SWs in the two materials. In magnonics we decide to use as a reference the phase velocity of the SW propagating perpendicular to the magnetization direction in the YIG film^45, 47^:

1 $$n=\frac{{v}_{YIG}}{v}=\frac{k}{{k}_{{\rm{YIG}}}}.$$

In our case we took *k*_YIG_ = *k*_in_, i.e., wavenumber of the SW emitted by the microstripe antennae. The ratio of *n* related to the incident and refracted waves (no reflected wave after IFDRL analysis) is considered, which according to (1) refers directly to the change of the magnitude of wavevector. SW interaction with anidots line will be discussed below using both IFDRL method and MS.

Rules known from optics: the causality principle and the conservation of the wavevector component tangential to the boundary/interface between two homogeneous media are used in the IFDRL analysis. We assume emission of plane SWs with wavefronts parallel to the microstrip antenna. In the linear approximation the dependence of the SW frequency *f* on the wavenumber *k* for SWs propagating at an angle $\phi$ with respect to the magnetic field direction in a homogeneous ferromagnetic film with a thickness *d* and a saturation magnetization *M*_*S*_ reads^46^:

2 $$f=2\pi \gamma {\mu }_{0}{[H(H+{M}_{S}[1-P{\cos }^{2}(\phi )+\frac{{M}_{S}}{H}P(1-P){\sin }^{2}(\phi )])]}^{1/2},$$

where $P=1-\frac{1-{e}^{-kd}}{kd}$. We assume here free boundary conditions for the dynamic magnetization components and neglect the exchange interaction because of a predominant contribution of the magnetostatic interaction at low frequencies in the considered relatively thick YIG films. In the calculations below we have taken a tabular value of the magnetization in 4.5 μm thick YIG film at room temperature, *M*_S_ = 0.139 × 10^6^ A/m, and a gyromagnetic ratio γ = 176 rad GHz/T^13^.

**Figure 5:**
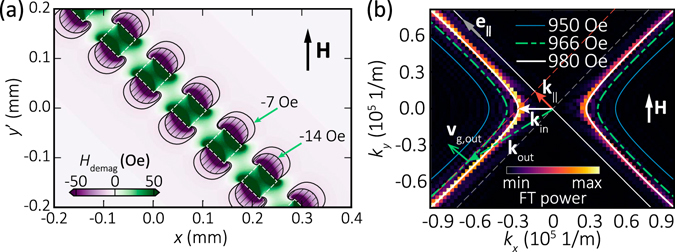
Modeling of spin wave interaction with the antidots line. (**a**) The demagnetizing field in the YIG film (4.5 μm thickness) near the square antidots row (of 50 μm size and 50 μm separation). The colormap shows the demagnetizing field component along the direction of the *H* oriented at the angle 45° with respect to the antidots line. (**b**) Iso-frequency dispersion relation lines at 4.7 GHz in the homogeneous YIG film for internal magnetic field magnitudes equal 950, 966 and 980 Oe with the thin blue solid lines, dash-dotted green and thick white solid, respectively. The direction of antidots line is marked as **e**_∥_. The wave vector of the incident and refracted SW is marked with **k**_in_ and **k**_out_, respectively, their component tangential to the line of andidots is *k*_||_. The group velocity direction of the refracted SWs is marked with **v**_g,out_. In the background by color-map is presented dispersion relation obtained using MS for the field magnitude equal 980 Oe. Perfect matching between simulations results and analytical contour is visible.

Let us now consider YIG sample corresponding to the experimental condition where the external magnetic field is 45° inclined from the antidots line. Figure 5(a) shows the calculated demagnetizing field appearing near the edges of the square antidots line in the ferromagnetic film^30^. The average internal magnetic field value is reduced from 980 to 966 Oe in an area in which the demagnetizing field changes gradually over a distance comparable to or smaller than the wavelength of the incident SWs λ_in_ (≈225 μm) determined for *H* = 980 Oe from Eq. (2). For SW interaction with the antidots line analysis IFDRLs were plotted over the (*k*_*x*_, *k*_*y*_) plane for a frequency of 4.7 GHz with *H* equal 950, 966 and 980 Oe, see Fig. 5(b). The IFDRLs are two open curves, symmetric with respect to the *x*- and *y*-axes, and with almost straight arms for *k* > 0.4 × 10^5^ 1/m.

We will explain SWs beam formation basing on a model assuming an interface between two values of the internal magnetic field *H*, 980 Oe and 966 Oe near the antidots line. According to the experimental configuration, the wavevector **k**_in_ (white arrow in Fig. 5(b)) of the incident SWs emitted by the microstrip antenna is oriented along the *x*-axis and at 45° with respect to the direction **e**_||_ of the antidots line (which is parallel to the asymptote of the iso-frequency curve for the discussed non-reflection effect case). The incident SWs, approaching the antidots line, are refracted at the interface between the regions of 980 and 966 Oe (dashed region in Fig. 5(a) pointed by −14 Oe). The condition *k*_in,∥_ = *k*_out,∥_ (see *k*_||_ being the component of the wavevector along the line of antidots, marked as red arrow in Fig. 5(b)) provides construction of the single wavevector **k**_out_ of the refracted SWs (dash-dotted green arrow in Fig. 5(b)). At the point indicated by the wavevector **k**_out_, the direction of the group velocity **v**_g_ of the refracted wave is defined by the normal to the IFDRL and is parallel to the antidots line direction **e**_∥_ (see **v**_g,out_ marked by green arrow in Fig. 5(b)). Due to hyperbolic character of IFDRL, there is no solution for reflected SWs fulfilling the condition *k*_in,∥_ = *k*_out,∥_ (for *k*_*x*_ > 0 required by the causality). Indeed, strong SW beam moving exactly along the line of antidots was observed in BLS experiment, see Fig. 3. Additionally one can deduce from Fig. 5(b) that |**k**_out_| > |**k**_in_|, i.e., the wavelength of the refracted SWs is significantly shorter than that of the incident SWs (at 966 Oe: λ_out_ = 2π/*k*_out_ = 65 μm). This wavelength reduction is similar to the effect of SW wavelength reduction appearing during the wave enetring into a medium with different refractive index driven by the thickness changes of the permalloy film^47^. We note that the described SW beam formation is a combined effect of the wave phenomena existing due to hyperbolic IFDRL of the magnetostatic spin waves and change of the SW refractive index near antidots resulting from the decreasing internal magnetic field, and it is different from the edge modes existing in the magnonic crystals^48, 49^.

The analytical IFDRLs were compared with the dispersion lines obtained from MSs, as can be seen in Fig. 5(b) good agreement was achieved, which validates presented approach. Strong SW beam creation will be further analyzed using MS in the following part.

**Figure 6:**
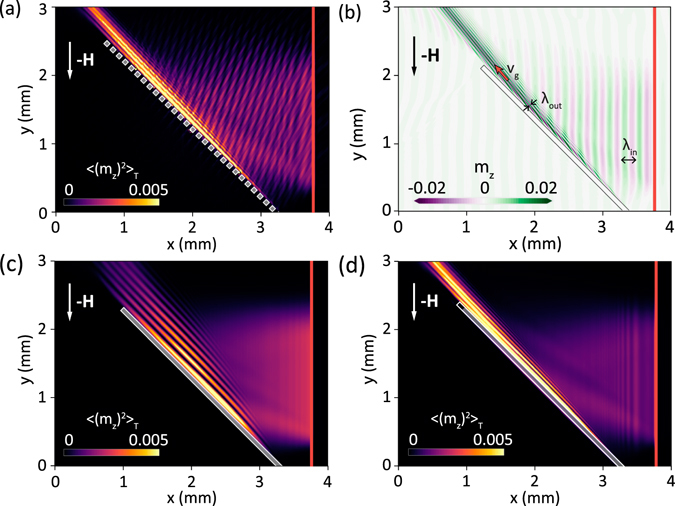
Results of MSs showing SWs interaction with: (**a**) antidots line of size 50 μm and period *a* = 100 μm at 4.7 GHz, (**b**) long rectangular hole (50 μm width) at 4.7 GHz, (**c**) long rectangular hole (50 μm width) at 4.98 GHz, and (**d**) on the area with the inhomogeneous internal magnetic field at 4.7 GHz. Note that in (**b**) SWs amplitude (i.e. the out of plane component of the reduced magnetization *m*_*z*_ = *M*_*z*_ */M*_S_) and in (**a**,**c**) and (**d**) SWs intensity are presented. The SWs are excited by the microstrip antenna (red rectangle) located on the right side of the antidots line and propagate on the left side of the antenna. The static magnetic field 980 Oe for (**a**–**d**) was oriented along the *y*-axis. In (**b**) we marked SW wavelengths of ingoing and outgoing SWs and group velocity of the outgoing SW beam.

We used MS (see Methods for technical details) to show SWs interactions in two patterned structures: (i) a sequence of square antidots with a 50 μm side and a 50 μm spacing and (ii) a long rectangular hole. Both patterns are aligned at the critical total non-reflection angle with respect to the direction of *H* (Fig. 6). θ_cr_ equals 45° and 980 Oe external magnetic field amplitude was chosen. The SWs were excited by a monochromatic microwave magnetic field at a frequency of 4.7 GHz; their spatial profile along the *x*-axis was obtained from the Biot-Savart law applied to the microstrip line being placed on the top of the YIG film with a homogeneous distribution of the microwave current. To avoid the excitation of SWs directly in the antidot area, the excited SWs had wavefronts parallel to the *y*-axis and were ca. 2 mm long. Nonreciprocal excitation by the microstrip antenna^15, 18^ was obtained by including the *z* component of the microwave magnetic field in the simulations. This component oscillates in antiphase on the two sides of the antenna, and is responsible for the changing of the SW propagation direction on reversal of the magnetic field^43^. The difference of SW intensities between SWs emitted on opposite sides of the microstripe obtained in MS is similar to the measured values, and reach 10, according with the literature data^41, 44^. The result of simulations, a high-intensity narrow SWs beam propagating along the line of antidots, with an amplitude much larger than that of the incident plane SW is visible in Fig. 6(a). The creation of strong SW beam in simulations is consistent with BLS experimental results shown Fig. 3. The wavelength of the refracted SWs is drastically shortened (λ_out_ = 70 μm, see Fig. 6b) with respect to that of the incident waves, and the wavevector is approximately perpendicular to the group velocity (which is parallel to the line of antidots).

Similar results of simulations were obtained for SWs scattering from a long rectangular hole, see Fig. 6(b), which shows almost the same features as in Fig. 6(a) for the antidots line. Reflections of SWs from the long rectangular hole are completely absent and there is no interference pattern on the right side of the row. That is different as compared to antidots line, where a slight pattern has been found (Fig. 6a). The interference pattern in Fig. 6(a) is a result of the nonuniformity of the demagnetizing field along the antidots line seen in Fig. 5(a) which causes some reflections of SWs. To ultimately verify the crucial role of the magnetic field inhomogeneity in the observed SW beam formation we simulated SW propagation in a homogeneous YIG film with an artificially introduced nonuniform internal magnetic field. For the internal field taken from the long rectangular hole simulations, we observed (Fig. 6d) almost the same behavior of the SWs, as in the case of the physical row – compare with Fig. 6(b). This leads to the conclusion that the experimentally observed total non-reflection and strong SW beam formation are caused by the inhomogeneous internal magnetic field rather than directly by the edge of the ferromagnetic film. Nevertheless, the edge of the sample is the source of the demagnetizing field there.

We have also checked numerically influence of the increasing SW frequency on the refracted wave. We found, that the increase of the frequency results in the rotation of the refracted SW beam towards the direction of the magnetic field. The exemplary result of MS at frequency 4.98 GHz is shown in Fig. 6(c), we clearly see a change of the refracted SW beam direction and its broadening with respect to the low frequency SWs (Fig. 6a). This dependence was confirmed also experimentally by increasing *f* to 5.2 GHz, and observing respective change of the beam direction. This can add additional functionality to our device, it is, the possibility to change a direction of the beam propagation with change of the frequency or alternatively with change of the external bias magnetic field magnitude, which shifts up or down the dispersion relation of SWs.

Finally, let us discuss the difference between the measurements and the MSs. In the simulations we have assumed a tabular value of *M*_S_, which may differ from the actual value in the studied sample. According to Eq. (2) an increase in *M*_S_ by less than 3% will shift the dispersion relation to higher frequencies, close to the experimental value. This explains the difference in frequencies used in simulations and measurements.

In summary, we demonstrated the application of the total non-reflection effect on the line of antidots in a few micrometers thick YIG film for construction of strong SWs beam switchers in the two geometries. For control of the direction flow of the SW beam we used the effect of non-reciprocity of SW excitation by a microstrip antenna. The results of SW interaction with the line of antidots studied by Brillouin light scattering spectrometry are consistent with the micromagnetic simulations and SW analysis by the iso-frequency curve method. The physical phenomenon allowing to obtain highly focused beams, originates from the refraction of plane SWs in the area of inhomogeneous internal magnetic field near the edge of either the antidots line or the long rectangular hole. A refracted strong SWs beam with a wavelength 3.5 times shorter than that of the incident plane waves can be obtained by reducing the internal magnetic field by 14 Oe on average. The wide frequency range (approximately 0.3 GHz) in which the effect is observed suggests a wideband operability of the SW switchers.

An alternative method of SW switching could be based on construction of the two antennas on the same YIG film: one laying over magnetic film and second one below. In that set-up, as was confirmed with additional MSs, a change of strong SW beam direction can be realized by a change of antenna exciting SWs at constant direction of the external magnetic field. New available technology of fabrication of low damping very thin films (e.g. submicrometer thickness YIG films) opens opportunities for desirable miniaturization of the proposed SW switchers. The dispersion relation of MSSWs involves *kd* (see, Eq. (2)) which means that decreasing the film thickness 100 times leads to the same properties, but for 100 times larger wavenumbers, i.e., for SWs of 100 times shorter wavelengths; the size of antidots can be decreased as well. However, further investigations are required for that demonstration, which will take into account the effect of the exchange field and respective modification of the iso-frequency dispersion li﻿nes.

## Methods

### Brillouin light scattering

The measurements were performed at room temperature. The microwave power was set to 5 dB, which was low enough to avoid any non-linear effects. The SWs scattered on the line of antidots were detected by space-resolved BLS spectroscopy in the reflection configuration. This method is based on the inelastic scattering of magnons with light from single frequency 532-nm solid-state laser focused on the sample surface. The frequency shift of the inelastically scattered light was analyzed using a six-pass Fabry-Perot interferometer JRS Scientific Instruments. The probe laser beam was scanned across the sample. The BLS intensity, which is proportional to the square of the dynamic magnetization amplitude, was recorded at various positions with step sizes of 0.015 mm along the *x* and *y* axes^39^.

### Micromagnetic simulations

MSs were prepared by means of open-source GPU accelerated MuMax3 environment^50^ which solves full Landau-Lifshitz equation using finite difference method in time domain. MSs were used in order to calculate the demagnetizing field profiles, the IFDRL and, finally, SWs excitation by the microstrip antenna and their refraction in the region of the patterned YIG film. In all MSs large systems were simulated, discretized using cubical cells of size: 2 μm × 2 μm × 1.5 μm. The width and length of simulated region were depended on a particular simulation demands and have varied from 2 mm up to 5 mm. Additionally periodic boundary conditions along *x* and *y* axes were assumed. The damping parameter was set as α = 0.0001. All simulations were preluded by static simulations and, then, with stable magnetic configuration the simulations of the SWs dynamics were performed during which SWs were excited by dynamic magnetic field and propagation of the SWs was studied. In simulation we assumed *M*_S_ = 0.139 × 10^6^ A/m, γ = 176 GHz/T and the exchange constant *A* = 0.4 × 10^−11^ J/m.

#### IFDRL simulations

IFDRLs were obtained according to ref. 51. The size of studied structure was 2 mm × 2 mm × 1.5 μm and sampling interval was set as 50 ps. SWs were excited using dynamic magnetic field **B** = 0.001sinc(*k*_cut_ *ρ*)sinc(2π*f*_cut_ *t*)**e**_*z*_ where ρ is the distance from the excitation center, *k*_cut_ = 0.5 × 10^6^ 1/m and *f*_cut_ = 15 GHz. At the first stage two-dimensional SWs dispersion relation |*M*(*f*, *k*_x_, *k*_y_)| was obtained, and afterwards one particular frequency *f*_0_ was chosen. In the presented in Fig. 5(b) IFDRL it was *f*_0_ = 4.7 GHz.

#### SWs excitation

The SWs were excited by the dynamical external magnetic field *H*_exc_(*t*, *x*, *y*) = *G*(*y*)*H*_a_(*x*, *y*)sin(2π*f*_0_ *t*) with the frequency *f*_0_ = 4.7 GHz. The magnetic field profile *H*_a_(*x*, *y*) was obtained using Biot-Savart law for the 30 μm width microstrip antenna placed on the top of YIG film, 1 μm separation between antenna and YIG film was assumed. The field profile *H*_a_(*x*, *y*) was normalized to the value of 10 Oe. Additionally, in order to simplify results analysis, additional Gaussian-like envelope *G*(*y*) with amplitude value varying along the *x*-axis and normalized to 1 was introduced.
